# Radial scar on image-guided breast biopsy: is surgical excision necessary?

**DOI:** 10.1007/s10549-018-4741-y

**Published:** 2018-03-12

**Authors:** Wendy Yen Yun Chou, Deborah J. Veis, Rebecca Aft

**Affiliations:** 1 Washington University in St. Louis, 660 South Euclid Avenue, St. Louis, MO 63110 USA; 2 Department of Pathology and Immunology, Washington University School of Medicine, St. Louis, MO USA; 3 Department of Surgery, Washington University School of Medicine, St. Louis, MO USA; 4 John Cochran Veterans Administration Hospital, St. Louis, MO USA

**Keywords:** Breast cancer, Radial scar, Core needle biopsy

## Abstract

**Purpose:**

Radial scar’s stellate appearance may mimic carcinoma mammographically and histologically. Management of radial scar (RS) found on breast core needle biopsies (CNB) ranges from excision to clinical observation due to the variation in reported upgrades to malignancy at surgical excision. We examined the upgrade rate in patients with RS detected on CNB at our institution and reviewed the current literature.

**Methods:**

A retrospective study was conducted of all cases with RS diagnosed on CNB between December 2006 and March 2017 at our institution. Inclusion criteria were patients with “pure” RS and RS associated with high-risk lesions (HRL). Upgrade was defined as invasive or non-invasive cancer in the excisional biopsy.

**Results:**

157 cases were identified with RS on CNB, and 122 cases met inclusion criteria. Of these 122 cases, 91 (75%) had pure RS on CNB while 31 (25%) had associated atypia or HRL. 81 (66%) of patients proceeded to excisional biopsy and 41 (34%) did not. Two patients (1.6% of total) were found to have a low-grade invasive ductal carcinoma (0.6 and 0.8 cm) upon surgical excision. None of the remaining 120 patients developed an ipsilateral breast cancer with a mean of 32.3-month follow-up.

**Conclusions:**

We found a very low upgrade rate to breast cancer when RS was found on CNB with or without associated HRL. Our results are consistent with other reported series. Our data do not support surgical excision for RS but rather close clinical follow-up for patients with RS on CNB.

**Electronic supplementary material:**

The online version of this article (10.1007/s10549-018-4741-y) contains supplementary material, which is available to authorized users.

## Introduction

Radial scars (RS) are entities which are described mammographically and pathologically. They are benign breast lesions that are commonly detected by pathologists [1]. The risk of subsequent breast cancer associated with RS found on pathology with and without atypia ranges from 1.1–3.0 to 2.8–6.7% respectively [2]. Patients with RS in benign breast biopsies were found to have twice as great risk of subsequent breast cancer [1, 3].

Radiographically, RS are characterized by an area of architectural distortion with a central radiolucency and the presence of radiating spicules. RS, detected on breast pathological specimens, are benign lesions and are part of the sclerosing adenosis spectrum. Histologically, RS are characterized by a central fibroelastic core containing entrapped glandular elements and ducts that radiate outward giving the lesion a characteristic stellate appearance. Histologic and radiographic RS do not have an exact correspondence [4–6] and the majority of pathological RS are found incidentally at biopsy for unrelated mammography findings. Because of the reported association of RS with surrounding proliferative disease and malignancy, excisional biopsy of RS detected on core needle biopsy (CNB) has been advocated [7–9].

Several histological diagnoses found at image-guided CNB of breast lesions have been shown to be associated with a sampling error rate that is high enough to warrant surgical excision. RS found on CNB from image-guided biopsies may be a microscopic incidental finding, may account for the imaging findings, or may be a high-risk lesion warranting surgical excision. Several retrospective reviews of surgical specimens obtained after RS diagnosis on CNB have reported an increased incidence of associated proliferative disease, malignancy, or both [7, 9–15]. Some studies have reported malignancy rates associated with RS with surgical excision as high as 29–30% [9, 16, 17]. Based on the association of RS with other proliferative or malignant processes or the possibility of sampling error, surgical excision has become the standard of care to avoid missing a low-grade cancer or upgrading to a high-risk lesion.

Given the improvement in imaging, biopsy devices, and pathological examination, we examined the outcome of patients with RS detected on CNB at our institution as well as reviewed the current literature.

## Methods

This retrospective study was reviewed and approved by the Institutional Review Board of Washington University School of Medicine. The Barnes-Jewish Hospital pathology database was searched for the terms “RS” and “RS core” from December 2006 to March 2017. Inclusion criteria included patients with “pure” RS and RS associated with high-risk lesions (HRL). Both imaging-targeted lesions and incidental pathologic findings of RS were included. Exclusion criteria were RS associated with malignancy at percutaneous biopsy, patients with simultaneous or previous ipsilateral breast cancer diagnosis within 6 months prior to CNB, patients who were lost to follow up as defined by last follow-up date corresponding to the date of the CNB, and patients who were never physically seen at our medical center, though their pathology was reviewed at our institution. Follow-up was defined as the time interval from the CNB to the date of the last recorded visit to our institution.

All mammograms were interpreted and biopsies recommended by a core of five radiologists who are dedicated fellowship trained mammographers. All pathology results were interpreted by dedicated breast pathologists. Each CNB was recorded for its modality [stereotactic, ultrasound (US), or MRI-guided], mammographic finding, and excisional pathology when performed. Ipsilateral atypia or carcinoma in the same core biopsy was also recorded. High-risk lesions (HRL) included atypical ductal hyperplasia (ADH), lobular carcinoma in situ (LCIS), atypical lobular hyperplasia (ALH), and papilloma. In cases where there was more than one HRL in the biopsy, the higher ranked HRL was recorded. For our study, an upgrade from the CNB was defined as upgrade to an invasive or non-invasive breast cancer on the excisional pathology report. Benign lesions were defined as any lesion that did not require further clinical treatment.

### Statistical method

Tests for statistical significance of the association between demographic/clinicopathologic factors and pathologic upgrade were performed using the Chi square (*χ*^2^) test. Statistical significance was defined to be *P* < 0.05.

## Results

RS was identified on 157 image-guided CNB. 35 cases were excluded from this study due to simultaneous ipsilateral breast cancer, lost to follow up, or never seen at our medical center (Supplemental Table 1). Of the remaining 122 patients, approximately one-third of the women were between the ages of 50 and 69. Approximately 80% were Caucasian and 20% minority, reflective of the population served by the medical center. The most common mammographic findings for biopsy recommendation were architectural distortions (27%), mass (22%), and calcifications (19%). Mammographic mass diameter measured from the mammographic or US views ranged from 0.3 to 3.5 cm (mean 0.9 cm). The most common types of diagnostic biopsies performed were stereotactic core vacuum-assisted biopsy (VAB) with 9-gauge needles (67%), followed by US-guided biopsy with 14-gauge needles (24%) and MRI-guided biopsy with 9-gauge needles (9%) (Table 1).

**Table 1:** Demographics and clinicopathologic features of 122 patients with RS on breast core biopsy

Characteristic	Patients who underwent surgical excision, *n *= 81 (%)	Patients who did not undergo surgical excision, *n *= 41 (%)	*P* value
Age—*n* (%)			0.02*
< 39	4 (5)	0 (0)	
40–49	15 (19)	6 (15)	
50–59	30 (37)	7 (17)	
60–69	22 (27)	13 (32)	
70–79	8 (10)	12 (29)	
> 80	2 (2)	3 (7)	
Age—mean (SD)	58 (10)	64 (12)	
Race/ethnicity—*n* (%)			0.09
White	71 (88)	32 (78)	
Hispanic	0 (0)	0 (0)	
Asian	2 (2)	1 (2)	
African-American	4 (5)	8 (20)	
Other	2 (2)	0 (0)	
Unknown	2 (2)	0 (0)	
Indication for core biopsy—*n* (%)			0.10
Architectural distortions	26 (32)	7 (17)	
Architectural distortions + calcifications	10 (12)	3 (7)	
Architectural distortions + mass	8 (10)	1 (2)	
Architectural distortions + focal asymmetry	0 (0)	1 (2)	
Mass	17 (21)	10 (24)	
Mass + calcifications	0 (0)	2 (5)	
Asymmetrical density	1 (1)	0 (0)	
Focal asymmetry	4 (5)	4 (10)	
Focal asymmetry + mass	1 (1)	0 (0)	
Calcifications/microcalcifications	12 (15)	12 (29)	
Non-mass like enhancement	2 (2)	1 (2)	
Type of core biopsy—*n* (%)			0.47
Stereotactic	52 (64)	30 (73)	
Ultrasound	22 (27)	7 (17)	
MRI-guided	7 (9)	4 (10)	
Biopsy needle gauge (G)—*n* (%)			0.84
9 G-vacuum assisted	74 (91)	37 (90)	
14 G-spring assisted	7 (9)	4 (10)	

*Statistically significant *P *< 0.05

81 (66%) of 122 patients underwent a surgical excision of the RS area and 41 (34%) did not. Seven of the patients (17%) who did not undergo an excision were diagnosed with a concurrent contralateral breast cancer and the RS was diagnosed during their work-up. The mean age of those undergoing excision was 64 versus 58 for those who did have further surgery and this was significant (*P *= 0.02). Otherwise, there was no significant difference in race, mammographic indication for core biopsy, type of core biopsy, and biopsy needle gauge between those patients who had an excision versus those who did not. There were a slightly higher percentage of African-American women who did not undergo surgical excision compared to African-American women who underwent surgery (19 vs. 5% respectively) (Table 1).

### RS with atypia on core biopsy

**Fig. 1:**
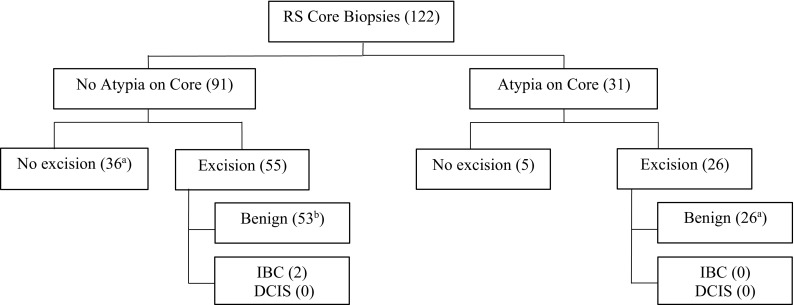
Outcome of patients diagnosed with RS on CNB (number). Number of cases with contralateral breast cancer: ^a^7 cases, ^b^4 cases

Of the 122 core pathologies reviewed, 91 (75%) were not associated with atypia and 31 (25%) were associated with atypia, including ADH, ALH/LCIS, and papilloma (Table 2). Of the 31 patients with RS and atypia on image-guided CNB, 26 (84%) of the patients had an excisional biopsy and none of these patients were diagnosed with a breast cancer on the excisional biopsy (Fig. 1). None of the five patients who did not have an excisional biopsy (all with RS/papilloma on CNB) were later diagnosed with an ipsilateral breast cancer with a mean follow-up of 75.0 months (range 0.4–111.6 months) nor were any of the patients who had an excisional biopsy diagnosed with an ipsilateral breast cancer with a mean follow-up of 23.4 months (range 0.5–94.0 months) (Table 3).

**Table 2:** Core needle biopsy pathology (*n *= 122)

Core biopsy results	*n* (%)
“Pure” radial scar	91 (75)
Radial scar with ADH	9 (7)
Radial scar with lobular neoplasia	8 (7)
Radial scar with papilloma	14 (11)

**Table 3:** Surgical pathology of radial scars (*n *= 81)

Biopsy pathology	*n* (%)	Surgical pathology
“Pure” radial scar	55 (68)	2 IDC
Radial scar with ADH	9 (11)	All benign
Radial scar with lobular neoplasia	8 (10)	All benign
Radial scar with papilloma	9 (11)	All benign

### Pure radical scar

Of those 91 patients with RS only on core biopsy, 55 (60%) underwent an excisional biopsy and 36 (40%) did not (Fig. 1). Of those 36 patients who did not undergo an excisional biopsy, none of the patients developed an ipsilateral breast cancer with a mean follow-up of 42.9 months (range 0.1–101.2 months). Of the 55 patients without atypia who did undergo surgical excision, 53 (96%) of the pathologies were benign and 2 cases yielded small invasive cancers. Both cancers were invasive ductal carcinoma (IDC), grade 1, estrogen receptor-positive/Her2-negative and were 0.6 and 0.8 cm, respectively (Table 4).

**Table 4:** Cancer upgrade cases after surgical excision

Patient age	Image type	Indication for biopsy	RS pathology	Biopsy method	Biopsy needle gauge	Cancer type	ER/PR/Her2
59	Mammogram	Architectural distortion, mass (8 mm)	RS	US-guided spring assisted	14 G	IDC, grade 1, 6 mm	ER+/ PR+/ Her2−
63	Mammogram	Mass (7 mm)	RS fragments	US-guided spring assisted	14 G	IDC, grade 1, 8 mm	ER+/ PR+/ Her2−

### Contralateral breast cancer

In our study, 18 patients had concurrent contralateral breast cancer. Of these patients, 11 had pure RS found on CNB, while 7 had associated atypia on CNB. Patients with associated atypia on CNB all underwent surgical excision and all of the pathologies were benign. Of the patients with pure RS found on CNB, 4 underwent excision with resultant benign pathology, while 7 underwent imaging follow-up (mean 49.3 months, range 0.7–101.2 months). The mean follow-up for this group was 39.9 months, and none of the patients developed an ipsilateral breast cancer. All received appropriate treatment for their contralateral breast cancer.

## Literature review

We reviewed 16 studies (including our data) published from 2014 to 2018 with chart reviews from 1994 to 2017 (Table 5). For each study, the number of excisions performed after pure RS was found on initial CNB, and the number of upgrades to carcinoma, both invasive and non-invasive, and the type of carcinoma [DCIS or Invasive Breast Cancer (IBC) which includes IDC and invasive lobular carcinoma (ILC)] were recorded. An overall upgrade rate among the studies was calculated. From our literature review, there were a total of 37 out of 1085 cases of upgrades to carcinoma yielding an overall upgrade rate of 3.4%, which is consistent with our institutional study.

**Table 5:** Literature review

Year	Author	Upgrade rate (%)	Excision (*n*)	Upgrade (*n*)	DCIS (*n*)	IBC (*n*)
2014	Miller et al. [24]	2.0	102	2	1	1
2015	Matrai et al. [25]	0.0	77	0	0	0
2015	Conlon et al. [21]	0.0	47	0	0	0
2015	Nassar et al. [18]	10.5	38	4	2	2
2016	Leong et al. [20]	0.6	161	1	1	0
2016	Donaldson et al. [26]	0.0	37	0	0	0
2016	Mooney et al. [3]	16.0	25	4	3	1
2016	Kalife et al. [27]	2.4	41	1	1	0
2016	Park et al. [28]	0.0	10	0	0	0
2016	Li et al. [22]	0.9	220	2	1	1
2016	Hou et al. [29]	0.0	40	0	0	0
2016	Kim et al. [23]	1.6	63	1	1	0
2016	Rageth et al. [2]	10.9	46	5	4	1
2017	Nakhlis et al. [30]	8.8	34	3	2	1
2017	Ferreira et al. [19]	13.5	89	12	7	5
2018	Chou et al.	3.6	55	2	0	2
Total		3.4	1085	37	23	14

Of the 15 other studies, four studies: Nassar et al. [18], Mooney et al. [3], Rageth et al. [2], and Ferreira et al. [19] yielded comparatively high upgrade rates (10.5, 16.0, 10.9, 13.5%, respectively, average upgrade rate 12.6%, 25 of 198 total patients) to malignancy compared to the other 11 studies (average upgrade 1.4%, 12 of 887 patients). The exclusion criteria for first three studies were similar in that patients with any atypia observed with RS on CNB were excluded. Nassar et al. also excluded cases in which the pathological diagnosis on the CNB was RS (≤ 1.0 cm) or Complex Sclerosing Lesions (CSL) (> 1.0 cm) associated with atypical epithelial hyperplasia, lobular neoplasia, DCIS, and malignancy [18]. Mooney et al. excluded RS associated with any type of epithelial atypia and cases with known synchronous ipsilateral breast cancer [3]. Ferreria et al. excluded patients with a simultaneous breast malignancy (invasive or in situ breast cancer) and also patients with a follow-up interval less than 12 months if surgery was not performed after an image-guided CNB [19]. Rageth et al. did not specifically list exclusion criteria in their publication [2]. Whereas the exclusion criteria of the remaining 11 studies were all patients with concurrent ipsilateral breast cancer, coexisting atypia present with RS in core specimen, excision at an outside institution, lost to follow up after benign pathology results, and/or radiology–pathology discordance [20–30]. Thus, variability in exclusion criteria may account for the higher upgrade rates in some studies compared with others.

## Discussion

It has been recommended that patients with RS detected on image-guided CNB undergo surgical excision due to the high upgrade rate observed in surgical specimens. In our institutional series, we observed a low upgrade rate of 3.6% for all patients who had a surgical excision after CNB. Consistent with our finding, review of literature published between years 2014 and 2018, yielded an overall upgrade rate of 3.4%. Moreover, with a mean follow-up of 32.3 months, none of the other 120 patients in our series developed IBC or DCIS in the area of the RS biopsy, with or without excision.

A distinction needs to be made between RS detected mammographically versus that detected histopathologically. RS detected mammographically have been reported to be associated with early breast cancer, DCIS, and ADH in up to half of women and thus should be sampled by image-guided biopsy [4]. This is in contrast to RS detected on core biopsies for abnormal mammographic findings.

RS found histopathologically on CNB have been associated with multiple mammographic entities including masses, architectural distortion, calcifications, asymmetries, and may or may not correspond to RS detected mammographically [2, 8]. Our data and data reported in the literature indicate that there is no single radiographic or mammographic finding associated with upgrade of RS found on CNB to a cancer or which identifies those RS which need to be excised versus those which can be safely monitored [31, 32]. For example, in our series, the mammographic findings which triggered the initial CNB in the two cases upgraded to cancer were a mass and an architectural distortion. In other reported series, upgrade to cancers was associated with architectural distortions, larger masses (≥ 1 cm), calcifications, and older age [3, 22, 23]. Thus, the management of RS detected on CNB has ranged from surgical excision [33] or mammographic surveillance [4].

Part of the current RS management dilemma is the high variability of upgrade rates of pure RS among different institutional studies. In our review of 16 studies, including our data, published between 2014 and 2018, we calculated an overall upgrade rate to cancer of approximately 3.4% in patients diagnosed with RS on image-guided CNB with a range of 0–16.0%. While no single factor could account for the wide range in upgrade rate in the studies, several factors, such as year of the biopsy, biopsy device, and patient population, may affect the variability upgrade rate in the reported studies.

We found that more contemporary studies, with data from 2000 to 2017, reported very low upgrade rates. Kim et al. reported an upgrade rate on excision of 1.6% (1 out of 63 patients) [23]. Similar to our study, 25% of their patients did not undergo surgical excision and with a median follow-up of 26 months; none of the patients developed cancer. Leong et al. found an upgrade rate < 1% in 161 patients with pure RS [20].

The type of biopsy device on upgrade rate has been examined to determine whether larger core biopsy gauge or VAB led to an improved false-negative rate for cancer detection when radial scar is detected on CNB. If a VAB showed RS on histology, some believed surveillance to be sufficient [2]. Three studies reported no upgrade to malignancy if VAB or needle greater than 11 g is used [34–36]. These three studies had a combined patient population of 422, which represents about 22% of the patients in the studies we reviewed; thus, it is can be reasonably assumed that RS detected on CNB using large core gauge or VAB can be safely managed conservatively.

High variability in upgrade rates may be due to differences in inclusion criteria for each study, which can lead to case selection bias. For example, in studies reported by Hou et al. [29] and Li et al. [22], all patients with a history or family history of breast cancer or atypical proliferative lesions defined as flat epithelial atypia, atypical ductal hyperplasia, and lobular neoplasia were excluded. As a result, Hou et al. [29] and Li et al. [22] observed upgrade rates of 0.0 and 0.9%, respectively.

Variability can also be attributed to the method some studies chose to characterize RS. Matrai et al. [25] limited the study population to those RS with that were 0.5 cm or smaller. However, studies have included patients with RS ranging from 0.1 to 5 cm [25, 32, 35, 37].

Upgrade from RS to cancers may be incidental findings as has previously been discussed [20–22] and is likely the case with the two patients in our series. Most cancers detected on surgical excision for RS on CNB are low grade and prognostically favorable [16, 31, 38, 39]. In the literature review, 2.1% of patients had DCIS after excision for RS on CNB and 1.3% had IBC. Thus, these indolent cancers may have become apparent with close clinical follow-up and later detection would likely have little effect on overall patient outcome.

In our institutional study, none (0/26) of the RS with atypia on CNB were upgraded to malignancy upon surgical excision. This is in contrast to the higher number of upgrades to malignancy upon surgical excision with initial diagnosis of RS with associated atypia on CNB seen in other studies [3, 4, 20, 26, 35]. In two studies of RS and HRL on CNB, Leong et al. [20] found 6 of 54 (11%) cases of surgical pathology upgrades and Donaldson et al. [26] found 7 of 21 (33%) cases of upgrades to malignancy. Our low rate of upgrade in RS with HRL on CNB could be due to the low patient number, improved imaging, and biopsy devices.

Several studies published between 2007 and 2012 suggested surgical excision of pure RS on CNB, due to the association with malignancy [32, 37] and false-negative biopsy in RS [33]. Nizri et al. examined the practice management of RS detected on image-guided biopsies [40] and found that 40% of breast surgeons recommended selective excision based on imaging and pathological correlation, size of the lesion, and the presence of atypia on the pathology and 57% of the respondents recommended routine excision.

Considering the advancements in breast imaging, pathology, and multidisciplinary approaches to current RS management, along with our findings in this study, we believe that surgical excision is not necessary for RS without associated atypia on core needle biopsies. Instead follow-up imaging, a less invasive approach, appears appropriate given the low upgrade rates to malignancy and the low-grade cancers detected. This would save 97 patients a surgical procedure to detect 3 low-grade cancers, 2 of which are likely to be DCIS.

There are limitations to our study including single institution, retrospective patient population, and high volume practice with dedicated mammographers and breast pathologists, which may not be available at every institution. We were also unable to reliably obtain information on related family history of cancer. Additionally, as with any retrospective study, we are subject to impose our biases on our sample population and are also limited to the cases seen at our institution.

## Conclusion

We have found a low upgrade rate to cancer in RS and RS with HRL detected on image-guided CNB. Our results are consistent with other large institutional studies. Given the low upgrade rate to malignancy, we propose that upon finding small, incidental RS on CNB, subsequent surgical excision is not necessary unless there are other reasons for excision such as the presence of HRL or discordance with the mammographic findings. Instead, close clinical follow-up or routine imaging, a less invasive approach, is recommended.

## Electronic supplementary material

Below is the link to the electronic supplementary material.

Supplementary material 1 (DOCX 40 kb)
