# Supplementary material for: Radial scar on image-guided breast biopsy: is surgical excision necessary?
Source: Breast Cancer Res Treat. 2018 Mar 12;170(2):313–20. doi: 10.1007/s10549-018-4741-y (PMC5999183; doi:10.1007/s10549-018-4741-y)
Supplement: Supplementary file 1 — Supplementary material 1 (DOCX 40 kb) [file 10549_2018_4741_MOESM1_ESM.docx]

**Supplemental Table 1**. Patients excluded from data set (n=35).

| **Reason for Exclusion from Data Set** | **n (%)** |
| --- | --- |
| Lost to follow up | 5 (14) |
| Simultaneous/previous ipsilateral cancer diagnosis 6 months prior to breast biopsy | 28 (23 DCIS, 3 IDC, 2 ILC) (80) |
| Never seen at our medical center | 2 (6) |
